# Boerhaave's Syndrome Post-colonoscopy: When Emesis Conceals an Emergency

**DOI:** 10.7759/cureus.88383

**Published:** 2025-07-20

**Authors:** Qin Hou, Yunxia Zuo

**Affiliations:** 1 Anesthesiology, West China Hospital, Sichuan University, Chengdu, CHN; 2 The Research Units of West China (2018RU012), Chinese Academy of Medical Sciences, West China Hospital, Sichuan University, Chengdu, CHN

**Keywords:** boerhaave syndrome, comprehensive treatment, ct esophagography, post-colonoscopy, spontaneous esophageal rupture

## Abstract

Boerhaave syndrome, a rare yet life-threatening spontaneous esophageal rupture, is classically associated with forceful emesis. We present a case of a 48-year-old male with no prior esophageal pathology who developed vomiting, epigastric pain and dyspnea after undergoing an elective colonoscopy. Notably, the patient had experienced emesis following alcohol consumption the day prior to the colonoscopy procedure but did not disclose this history during the pre-procedural assessment. Initial assessments misattributed his symptoms to sedation-related nausea and post-procedural ileus, thereby delaying critical diagnostic imaging. The computed tomography (CT) combined with esophagography revealed pneumomediastinum and a 1.5 cm distal esophageal tear. Surgical pleural decortication was performed. The patient recovered after four weeks of multidisciplinary care.

## Introduction

Spontaneous esophageal rupture, also known as Boerhaave syndrome, is a transmural tear of the esophagus caused by abrupt increases in intraesophageal pressure (e.g., vomiting, retching) [1]. However, the clinical presentation of esophageal rupture is often non-specific, closely mimicking more common pathologies, such as pneumonia, spontaneous pneumothorax, myocardial infarction, pulmonary embolism, or other gastrointestinal tract pathologies [2]. Therefore, the diagnosis of spontaneous esophageal rupture is difficult and can easily be missed. However, delayed diagnosis is associated with a higher risk of complications and mortality (16%-51%) [3]. This report highlights systemic diagnostic oversight by procedural and anesthesia teams, emphasizing the need for enhanced post-procedural vigilance.

## Case presentation

**Figure 1:**
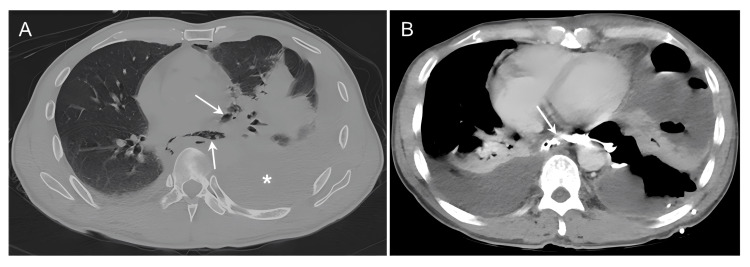
CT esophagography shows pneumomediastinum and perforation location. A, CT shows left hydropneumothorax (asterisk) and pneumomediastinum (arrow). B, CT scan with oral contrast agent (CA) revealed a fistula between the esophagus and the mediastinal paraoesophageal abscess (arrow).

A 48-year-old male patient underwent a scheduled colonoscopy due to the detection of positive fecal occult blood over the past two weeks. During pre-anesthesia assessment, he looked slightly nervous with a pulse rate of 98/min, blood pressure of 145/90 mmHg, respiratory rate of 20/min and SpO2 of 95% in room air. The patient's previous medical history was unremarkable except for an alcohol consumption (an average of 200 grams per week) for 10 years, therefore routine sedative and analgesic medications were intravenously administered with sufentanil 5 μg and propofol 70 mg. However, the colonoscopy was challenging and time-consuming, lasting approximately one hour, owing to inadequate bowel preparation. Five minutes after the procedure, the patient regained consciousness and complained of vomiting, epigastric pain and dyspnea. Sedation side effects, bowel perforation, pneumothorax, pulmonary embolism and acute myocardial infarction were initially considered. Physical examination revealed a soft abdomen with epigastric region tenderness and slightly weakened bowel sounds. Respiratory examination revealed tachypnea and weakened breath sounds on the left lung, and no signs of subcutaneous air and tracheal deviation were present. Cardiovascular examination displayed tachycardia with no murmurs. The bedside electrocardiogram demonstrated sinus tachycardia exclusively, with no evidence of waveforms suggestive of myocardial ischemic changes. Blood inflammatory indicators were significantly elevated (white blood cell count: 17.40 × 10^9^/L, reference range: 4-10 × 10^9^/L; serum procalcitonin: 55.65 ng/ml, reference range: < 0.05 ng/ml). The computed tomography (CT) esophagography revealed massive left hydropneumothorax and pneumomediastinum with gas tracking around the esophagus (Figure 1A), and bilateral pleural effusion with atelectasis and perforation of the lower left esophagus (Figure 1B).

Notably, the patient had experienced emesis following alcohol consumption one day ago but failed to disclose this history pre-procedure. We hypothesize that an esophageal rupture may have occurred prior to the procedure. Additionally, the patient had taken a bowel cleansing solution according to the guidance, potentially exacerbating the esophageal rupture and leading to severe infection.

Under direct visualization guided by X-ray, a 16-Fr nasogastric tube was successfully inserted. The patient was kept on nothing by mouth (NPO) status for two weeks. Besides, a comprehensive and integrated treatment strategy was implemented, encompassing fluid resuscitation, intensive nutritional support (primarily parenteral nutrition), intravenous antibiotic therapy, proton pump inhibitor (PPI) and the drainage of fluid collections. One week later, the patient underwent left pleural decortication, abscess clearance, and bilateral pleural drainage. After four weeks of the above treatment, he was vitally stable, and all his symptoms subsided, including his pain and dyspnea. An esophagram with contrast radiography revealed no significant abnormalities. Therefore, the nasogastric tube was removed. Then the patient started to intake clear fluid diet and gradually progressed to a soft food diet. After monitoring the improvement of the patient's health, he was allowed to be discharged on oral nonsteroidal anti-inflammatory drugs (NSAIDS), PPI and antibiotics. Two weeks post-discharge, the patient presented to the thoracic clinic for follow-up. He denied having any pain or difficulty in breathing. The CT esophagography revealed that the mediastinal emphysema and pleural effusion were completely resolved, with no evidence of contrast agent extravasation.

## Discussion

Spontaneous esophageal rupture is a rare (estimated incidence 1/6000), life-threatening emergency condition. Errors in diagnosis are usually caused by failure of clinicians to consider its possibility and unawareness of its varied and atypical presentations. Even a retrospective review reported vomiting was absent in 23% of spontaneous rupture of the esophagus [4], clinicians should consider the possibility of esophageal rupture in patients presenting with dyspnea and/or chest and abdominal pain following vomiting. CT scanning of the chest and abdomen, with water-soluble oral contrast, is recommended [5]. As illustrated by this clinical case, CT esophagography demonstrated both diagnostic efficiency and critical value in the identification of esophageal rupture.

Early diagnosis of esophageal rupture and rational treatment is lifesaving; that could be surgical or nonsurgical treatment [6,7]. Nonsurgical treatment includes aggressive management of sepsis, control of leaks, nutrition support and use of stenting, clipping, or vacuum therapy [8-12]. Previous reports suggested that, regardless of the time interval and sepsis, primary repair can be an option even in late esophageal perforation [13,14]. However, the patient in this case received decortication of the pleura, abscess clearance and drainage, instead of primary repair of the esophageal defect because the perforated site was too badly injured to realign due to severe infection. Fortunately, the patient achieved complete clinical recovery following multidisciplinary collaborative management.

## Conclusions

This case highlights Boerhaave syndrome as a life-threatening complication following colonoscopy, often concealed by nonspecific vomiting. Awareness of acute chest-abdominal pain after occult emesis is crucial for timely diagnosis. Early conducting CT esophagography can prevent fatal diagnostic delays. For patients presenting with severe sepsis, early primary repair poses significant challenges, thereby necessitating the prioritization of comprehensive multimodal therapeutic strategies. Clinicians should emphasize comprehensive pre-endoscopic history-taking and maintain a high index of suspicion for Boerhaave syndrome in post-procedural patients, even in the absence of overt vomiting. 
